# Automated Installation System for Joint Casing with Circumferential Temperature Control in District Heating Pipelines Using a Heat-Shrinkable PEX Tube

**DOI:** 10.3390/polym18070796

**Published:** 2026-03-25

**Authors:** Seungbeom Jang, Yuhyeong Jeong, Youngjin Jeon, Hyungsu Ju, Jooyong Kim, Yeonsoo Kim, Junghae Hwang, Dongil Choi, Jonghun Yoon

**Affiliations:** 1 Department of Mechanical Engineering, Hanyang University, Ansan-Si 15588, Republic of Korea; jangseung@hanyang.ac.kr (S.J.);; 2 Department of DH Network Administration, Pangyoro, Bundang-Gu, Seongnam-Si 13487, Republic of Korea; 3 Department of Mechanical Engineering, BK21 FOUR ERICA-ACE Center, Hanyang University, Ansan-Si 15588, Republic of Korea

**Keywords:** crosslinked polyethylene (PEX), heat-shrinkable polymer, DSC-defined thermal activation, thermal criteria, interfacial bonding, two-stage heating sequence

## Abstract

This study establishes experimentally grounded circumferential thermal criteria for heat-shrinkable crosslinked polyethylene (PEX) joint casings by coupling DSC-defined thermal activation with through-thickness thermal lag measured under trench-constrained irradiation. The activation temperature was identified as 140 °C from DSC, while an upper bound of the allowable outer-surface temperature was set to avoid thermal damage during installation. Full-scale temperature mapping revealed persistent circumferential non-uniformity caused by geometric line-of-sight limitations and inter-module gap regions, where the outer-surface temperature remained approximately 10–15 °C lower than directly irradiated locations, and the inner surface exhibited a delayed response due to the low thermal conductivity of PEX. Based on these observations, a two-stage heating sequence—an initial high-power stage followed by a reduced-power soaking stage—was experimentally derived to satisfy dual constraints: achieving inner-surface activation (≥140 °C) while maintaining the outer surface below the conservative outer-surface upper bound (~280 °C) and reducing circumferential temperature differences without surface overheating. Comparative joint tests confirmed that the proposed thermal criteria and sequence promote stable interfacial bonding and cohesive failure in the mastic layer, yielding higher repeatability and smaller strength scatter than conventional manual torch heating. The proposed framework provides experimentally grounded thermal criteria and a transferable procedure for designing heating conditions for heat-shrinkable polymer casing systems under constrained field environments.

## 1. Introduction

District heating (DH) networks distribute thermal energy from centralized plants to end users through extensive underground pipeline systems [1,2,3,4]. At field joints, adjacent steel service pipes are girth-welded, which requires local removal of the outer high-density polyethylene (HDPE) jacket and polyurethane-foam insulation [5]. The continuity of the external barrier is then restored by sealing the field joint with a heat-shrinkable casing [6,7,8,9]. Because localized sealing defects can initiate moisture ingress [6] and subsequent corrosion [10] and insulation degradation, the reliability of joint casing installation is critical for maintaining DH network integrity over the service life [6,10,11]. Therefore, establishing quantitative quality-assurance and quality-control procedures for casing installation is essential to ensure the long-term reliability of buried DH infrastructure [12,13].

In current field practice, heat-shrinkable casings are typically installed by manual gas-torch heating. However, trench geometry, adjacent-pipe clearance, and on-site accessibility vary from joint to joint, and the delivered heat flux depends strongly on operator-controlled parameters such as stand-off distance (heater–casing gap), heating time, and flame orientation. As a result, manual heating can readily produce circumferentially non-uniform surface heating with localized overheating and underheating. In addition, quantitative acceptance criteria for in situ installation are limited and are often complemented by operator judgment, which can lead to substantial joint-to-joint variability in installation outcomes [7,8,12]. Collectively, these access- and operator-induced variations motivate the need for a controlled and standardized heating strategy, potentially incorporating automation, to achieve consistent and verifiable installation quality under confined trench conditions. The heat-shrinkable casing investigated in this study is fabricated from semicrystalline crosslinked polyethylene (PEX), which is expanded during manufacturing and exhibits thermally activated shrinkage upon reheating [14,15,16,17,18,19,20,21]. Microstructurally, PEX retains stored deformation through crosslinking and crystallite constraints; when heated toward the crystalline melting endset region (approximately 140 °C in this study), reduced crystalline constraints and increased chain mobility allow the stored strain to relax, producing macroscopic circumferential shrinkage [22,23]. Subsequent cooling stabilizes the casing in the reduced-diameter state [15,16,17,18,19,20,21,22,23]. From a process-control standpoint, reliable installation requires not only sufficient outer-surface heating but also adequate through-thickness thermal penetration to activate the inner surface and the casing–mastic interface under thermal criteria that specify both a minimum activation condition and an upper bound for the allowable outer-surface temperature. Because PEX has a low thermal conductivity on the order of 0.3–0.4 W/(m·K) [22,24,25,26], through-thickness conduction is slow, and the inner-surface temperature can lag substantially behind the outer surface. In addition, in a typical field-joint assembly comprising the casing, mastic tape, and an outer HDPE jacket pipe, interfacial thermal resistance can further accentuate non-uniform temperature evolution across the composite thickness [27,28]. Consequently, the casing outer surface may reach the shrinkage activation range while the inner surface remains below the level required for complete shrinkage and sufficient contact pressure at the mastic interface, potentially degrading sealing performance. These characteristics necessitate a heating strategy with quantitatively specified thermal criteria that ensures through-thickness activation while mitigating circumferential temperature non-uniformity. Although automated induction- and infrared-based heating technologies have been reported for oil-and-gas pipelines, their implementation in buried DH environments is limited: induction heating cannot directly heat electrically insulating polymers such as PEX without additional susceptors, and infrared heating can be constrained by limited line-of-sight and clearance in narrow trench excavations [24]. Despite the widespread use of heat-shrinkable PEX casings in district heating pipelines, the installation process in field joints is still largely based on empirical heating practices using manual gas-torch heating, and the heating conditions are often determined by operator experience rather than by quantitative thermal criteria. In particular, circumferential temperature non-uniformity during heating and the through-thickness thermal lag between the outer and inner surfaces of the casing have not been systematically quantified, and reliable thermal criteria for ensuring sufficient activation of the casing–mastic interface while preventing surface overheating remain unclear. This lack of quantitatively defined thermal criteria makes it difficult to achieve consistent installation quality and to establish reproducible installation procedures.

To address these constraints, this study establishes experimentally grounded thermal criteria and a reproducible heating procedure for heat-shrinkable PEX joint casings under confined trench conditions. In this paper, the term heating procedure refers to the overall installation strategy governing circumferential heating progression and power modulation during joint installation, whereas heating sequence denotes the ordered set of operational steps used to implement this procedure. In other words, the heating procedure defines the overall heating strategy, while the heating sequence specifies the step-by-step operational execution of that strategy. The specific objectives are: (i) to characterize the thermal-transition behavior of the PEX casing and define a reference activation temperature using differential scanning calorimetry (DSC); (ii) to quantify circumferential non-uniformity and through-thickness thermal lag during full-scale external heating; (iii) to derive a two-stage heating sequence that satisfies the dual constraints of inner-surface activation and a conservative outer-surface temperature upper bound while mitigating circumferential non-uniformity; and (iv) to validate the proposed thermal criteria and heating procedure by benchmarking joint performance against conventional manual gas-torch heating, including shear testing and failure-mode analysis [6,7,9]. Although an automated installation apparatus is utilized to implement controlled circumferential heating in a narrow trench, the primary contribution is the transferable, criterion-based procedure for designing heating conditions for heat-shrinkable polymer casing systems.

## 2. Materials and Experiments

### 2.1. PEX Heat-Shrinkable Casing

**Figure 1:**
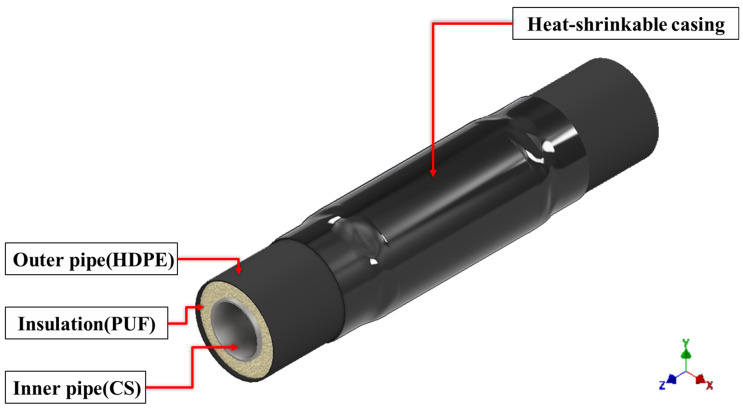
Schematic configuration of a pre-insulated district heating pipeline and the position of the heat-shrinkable casing at a welded joint.

In district heating (DH) systems, factory-made pre-insulated pipes are widely used and consist of a steel service pipe, a surrounding polyurethane-foam insulation layer, and an outer high-density polyethylene (HDPE) jacket that protects the insulation from mechanical damage and environmental exposure (Figure 1). During on-site installation or replacement, adjacent steel service pipes are connected by girth welding, which requires local removal of the outer HDPE jacket and polyurethane foam near the pipe ends. After welding, the exposed region must be re-insulated and sealed to restore the external barrier. In this study, the field joint was sealed using a heat-shrinkable joint casing installed over the welded region to bridge the gap between adjacent HDPE jackets [9]. When heated into its shrinkage activation range, the casing contracts circumferentially to conform to the joint geometry and applies compressive contact pressure that consolidates the mastic tape and forms an external seal. This field-joint sealing system is intended to provide resistance to moisture ingress and maintain mechanical robustness under service conditions, including thermal cycling and external soil loads [29].

**Figure 2:**
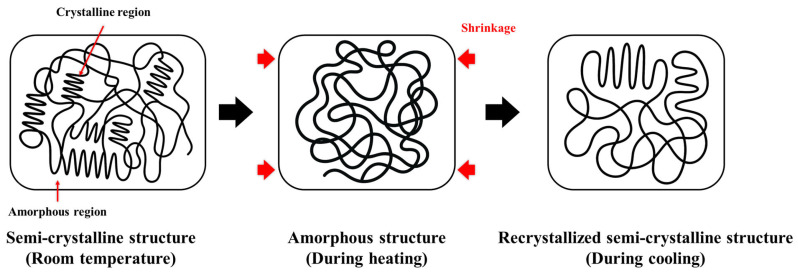
Schematic representation of the microstructural transition of PEX during the heat shrink process.

Figure 2 schematically illustrates the shrinkage mechanism of the PEX heat-shrinkable casing. During manufacturing, PEX is extruded as a flat sheet and stretched at an elevated temperature, typically along the machine direction, which induces chain orientation primarily in the amorphous phase and stores residual strain along the stretching direction [19,20,30]. The stretched sheet is then wrapped and seam-joined to form a cylindrical casing; consequently, the original stretching direction becomes the circumferential direction in the final product. At ambient temperature, crystalline domains impose physical constraints that maintain the expanded geometry, whereas the residual strain stored in the amorphous phase remains kinetically frozen until thermal activation. During field installation, reheating the casing into its shrinkage activation range reduces crystalline constraints and increases molecular mobility in the amorphous phase, enabling recovery of the stored strain toward a thermodynamically favorable random-coil configuration [19,20,21]. This recovery drives circumferential shrinkage and a reduction in casing diameter. Subsequent cooling suppresses further molecular rearrangements, thereby stabilizing the reduced diameter and maintaining shrinkage-induced interfacial compression under DH operating conditions. Overall, the shrinkage behavior of the PEX casing is governed by thermally activated strain recovery followed by fixation upon cooling [19,20,21], rather than by reversible thermal expansion and contraction. However, in the present study, this explanation is intended only as qualitative background for the observed shrinkage behavior, not as a quantitative material characterization.

**Figure 3:**
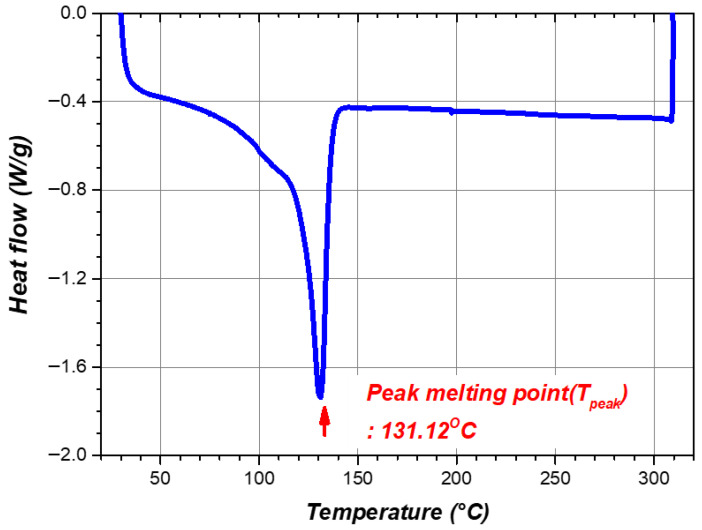
DSC result of heat-shrinkable PEX casing.

To characterize the thermal transitions relevant to shrinkage, differential scanning calorimetry (DSC) was performed on the PEX heat-shrinkable casing. DSC measures the heat-flow rate into or out of a specimen as a function of temperature at a prescribed heating rate, enabling identification of endothermic and exothermic events such as melting and crystallization. For heat-shrinkable semicrystalline polymers, DSC is particularly useful for identifying the melting transition of crystalline domains, which provides a practical temperature window for shrinkage activation. In this study, DSC scans were recorded from 30 °C to 310 °C at a heating rate of 10 °C/min, and the resulting thermogram is shown in Figure 3. Following ASTM E794 [31], the peak melting temperature ($T_{peak}$) was taken as the temperature at which the endothermic melting peak reaches its maximum. For the PEX casing, $T_{peak}$ was 131.1 °C. Because $T_{peak}$ corresponds to the maximum melting rate rather than completion of the melting process, the target shrinkage temperature should be selected closer to the melting endset temperature, defined here as the temperature at which the heat-flow signal returns to the post-melting baseline. Accordingly, $T_{act}$ was set to 140 °C based on the endset region of the DSC thermogram and used as the activation criterion in subsequent heating experiments. Although the DSC scan was extended to 310 °C to capture the full baseline under the instrument’s inert purge, joint installation is performed in ambient conditions. For the heat-shrinkable PEX casing considered in this study, the outer-surface temperature ($T_{outer}$) was limited to 280 °C as a conservative upper bound for the heating sequence, based on literature-reported high-temperature processing conditions for polyethylene-based materials [32].

In addition to the DSC-based activation-temperature definition, the first-heating DSC curve was also used to estimate the crystallinity of the PEX casing. The crystallinity was calculated using $X_{c}$ = Δ$H_{m}$/Δ$H_{\left(m,100\right)}$ × 100, where ΔH_(m,100)_ was taken as 293 J/g [33] for 100% crystalline polyethylene. Based on the first heating cycle, the melting enthalpy (Δ$H_{m}$) was approximately 71.0 J/g, corresponding to a crystallinity of about 24.2%. For additional material-related context, related literature reported a degree of crosslinking corresponding to a gel content of 81.0% and an elastic modulus-related E′ value of 584 MPa at 23 °C [34]. However, in the present study, these values were used only as reference data for interpreting the general behavior of PEX, not for direct quantitative characterization of the material state.

In addition to the DSC-based activation-temperature definition, the through-thickness heat-transfer behavior of the PEX casing can be approximated using a simplified one-dimensional heat-conduction model. To provide a first-order basis for interpreting the measured temperature difference between the outer and inner surfaces of the PEX casing, the casing wall is modeled as a one-dimensional plane wall of thickness, $t_{c}$. Let x denote the through-thickness coordinate (x = 0 at the outer surface and x = $t_{c}$ at the inner surface). The transient temperature distribution T(x,t) in the wall is governed by the one-dimensional heat-conduction equation:

(1) $$\rho c_{p}\frac{\partial T}{\partial t}\;=\;k\frac{\partial^{2}T}{\partial x^{2}}$$

where T(x,t) is the temperature within the casing wall, x, is the through-thickness coordinate measured from the outer surface, and t is time. The material properties are the density, ρ, specific heat capacity, $c_{p}$, and thermal conductivity, k, of PEX. In this simplified model, the characteristic timescale ($t_{char}$) for the inner-surface temperature ($T_{inner}$) to respond to heating at the outer surface scales as

(2) $$t_{char}\;\sim \;\frac{t_{c}^{2}}{\alpha }$$

where thermal diffusivity, $\alpha =k/(\rho c_{p})$*,* is a material property of the casing. Accordingly, for a given material, the time required for the inner surface to reach the activation temperature scales approximately with the square of the wall thickness, assuming comparable thermal boundary conditions. In multilayer casing–mastic–pipe configurations, interfacial thermal resistance further delays heat transfer to the mastic interface. As a result, the casing outer surface can reach the activation temperature earlier than the inner surface, motivating the need for carefully controlled heating conditions.

The above formulation is a simplified one-dimensional estimate introduced to illustrate the characteristic through-thickness thermal response of the casing wall. It is used only for qualitative interpretation of the observed thermal lag, not for rigorous quantitative prediction.

### 2.2. Joint Casing Installation in District Heating Pipelines

Leak-tight sealing at the casing–mastic tape and mastic tape–pipe interfaces is essential to preserve the joint’s thermal-insulation performance and to prevent moisture ingress. Achieving durable sealing requires (i) circumferentially uniform casing shrinkage that conforms to the joint geometry and (ii) sufficient thermal activation of the mastic tape at both interfaces. Here, mastic activation denotes the temperature range in which the mastic exhibits adequate softening, viscoelastic flow, and wetting so that, under shrinkage-induced compressive pressure, it can penetrate microscale surface irregularities and establish intimate contact, mechanical interlocking, and adhesion. These requirements motivate the standardized field installation procedure summarized below.

**Figure 4:**
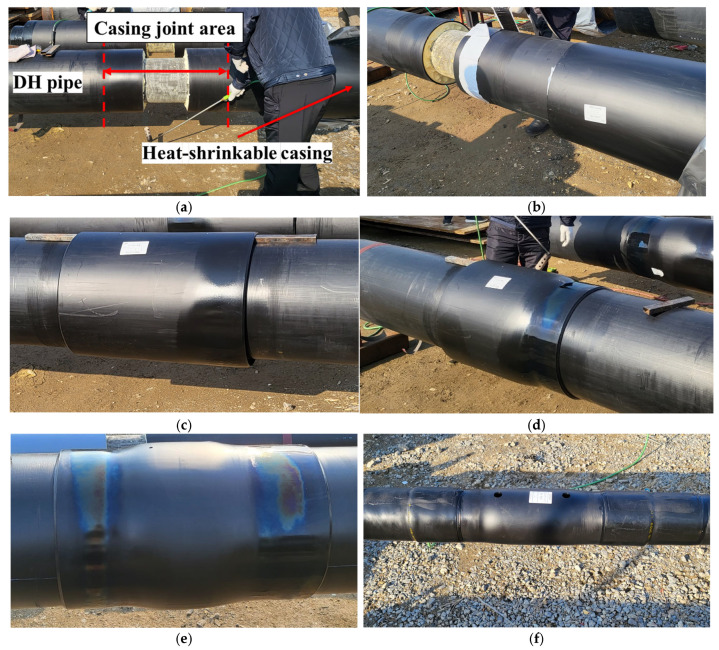
Joint casing installation process: (**a**) Steel service-pipe girth welding; (**b**) Mastic tape attachment; (**c**) Casing positioning and concentric alignment; (**d**) Bonding process with heat shrinkage; (**e**) Final shape of bonded casing; (**f**) Drilling and plugging process for insulation.

Figure 4 summarizes the step-by-step installation procedure for a heat-shrinkable casing, based on the construction guidelines of the Korea District Heating Corporation (KDHC) [9,30]. Prior to steel service-pipe girth welding (Figure 4a), the casing is slid onto one of the pipe sections. After circumferential welding of the steel service pipes is completed, the end surfaces of the HDPE jacket pipes are thoroughly cleaned to remove oxides and contaminants to ensure reliable adhesion of the mastic tape. The joint region is subsequently preheated locally to reduce initial temperature non-uniformity and to promote a uniform temperature rise during full-circumference heating. Figure 4b shows attachment of the mastic tape, which is specified to extend approximately 10 mm beyond the end of the heat-shrinkable casing. By providing a controlled overlap, this configuration reduces outward mastic extrusion during shrinkage and ensures robust sealing at the casing boundary. In Figure 4c, casing positioning and concentric alignment are achieved, and spacers are used to maintain concentricity between the casing and the pipe. Because the softened casing may sag under its own weight during heating, maintaining initial concentricity is critical to circumferential shrinkage uniformity and adhesion at the interfaces. Figure 4d illustrates the heat-shrinking process. Heating begins at the bottom (6 o’clock position) to create an initial bonded region and to prevent sagging by ensuring that the lower region adheres first. The heating zone is then expanded sequentially from the lower region to the middle and upper regions, thereby promoting uniform circumferential shrinkage. During heating, the PEX material softens and molecular mobility increases, which releases the stored circumferential residual strain. The resulting shrinkage force compresses the mastic tape against the pipe surface, promoting interfacial bonding. Figure 4e shows the final shape of the bonded casing. At this stage, a visual inspection is performed to check circumferential shrinkage uniformity, edge lift-off, unbonded regions, and geometric irregularities. Finally, Figure 4f shows the drilling and plugging step for subsequent injection of polyurethane foam insulation, which restores the thermal insulation performance of the joint. The preheating step is applied to reduce the initial temperature difference around the joint region before the main heating process. The bonding and shrinkage process is governed by the thermal activation of the PEX casing and the mastic layer. Based on the DSC analysis, the activation temperature of the PEX casing was identified as approximately 140 °C. Therefore, the installation process is controlled by the thermal criterion that the inner surface of the casing reaches the activation temperature while the outer surface remains below the degradation threshold. The effective heating duration is determined by the two-stage heating sequence developed in this study rather than a fixed holding time.

### 2.3. Shear Test for Bonding Strength

**Figure 5:**
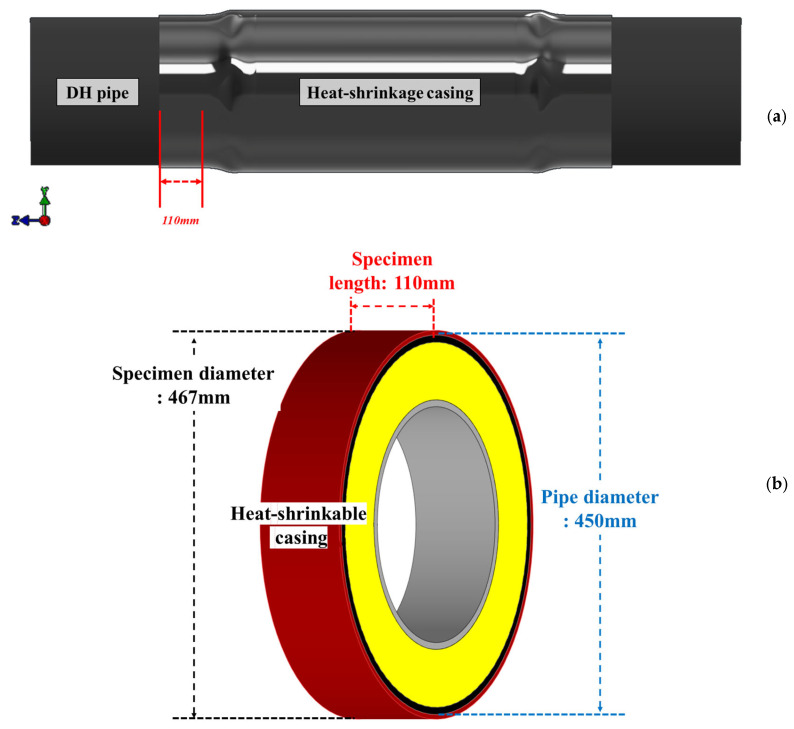
Shear test specimens were extracted from a DN 300 heat-shrinkable casing: (**a**) Bonded casing; (**b**) Shear specimen.

Comparative shear tests were performed to quantify the interfacial shear strength at the PEX casing–mastic tape interface for joint casings produced by conventional manual torch heating and by the proposed automated installation system. The tests followed DVS 2203-6 [35], enabling direct benchmarking of bond strength developed under the two heating approaches. Specimens were extracted from fully shrunk DN 300 heat-shrinkable joint casings [7], as illustrated in Figure 5. A 110 mm long ring section was cut from the central region of each casing to include the bonded PEX casing–mastic tape interface (Figure 5a). The resulting ring specimen (Figure 5b) had an outer diameter of approximately 467 mm, corresponding to the post-shrinkage diameter of a casing installed over a steel service pipe with an outer diameter of 450 mm. The specimen dimensions were selected to provide a sufficiently large bonded area for reliable measurement while remaining compatible with the push-out fixture and representative of an actual DN 300 field-joint casing.

**Figure 6:**
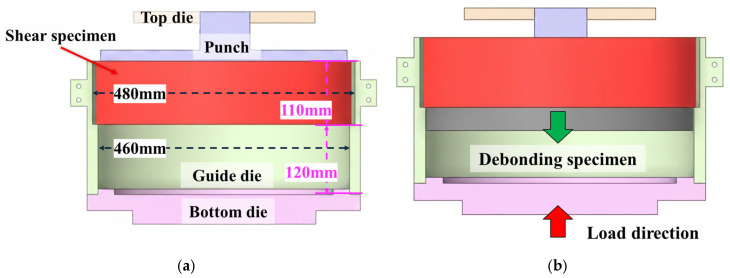
Shear test set-up: (**a**) Jig structure; (**b**) Schematic of loading motion during the shear test.

The experimental setup used to evaluate the interfacial shear strength is shown in Figure 6. Figure 6a presents the fixture geometry used for the shear test, which consists of a cylindrical punch and a guide die that supports the ring specimen and maintains coaxial alignment. The ring specimen was seated in the guide die with a 120 mm support length to constrain the outer circumference and suppress radial deformation during loading, and the overall fixture height was approximately 385 mm to accommodate the specimen geometry. Figure 6b illustrates the loading motion during the shear test: the specimen is supported by the guide die while the punch advances downward to apply compressive displacement to the inner steel-pipe segment, thereby driving relative sliding at the PEX casing–mastic tape interface. The fixture was mounted in a servo-hydraulic testing machine, and a vertical compressive load was applied at a constant crosshead speed of 5 mm/min. The load–displacement response was recorded and used to calculate the interfacial shear strength from the peak load divided by the nominal bonded area. Comparative shear tests were conducted on specimens prepared using the developed automated joint casing installation system and on specimens produced by the conventional manual gas-torch method to assess differences in interfacial bonding strength and repeatability. For each heating condition, three specimens were prepared and tested according to the DVS 2203-6 procedure. Because the experiment involves full-scale district heating pipe joints and installation equipment, the number of specimens was limited. The reported shear strength values correspond to the average of the three measurements.

## 3. Development of Automated Installation System

### 3.1. Design of Heating Modules

The heating module design was developed based on the geometric configuration of a DN300 district heating pipeline joint. The steel service pipe has an outer diameter of 450 mm, and the welded joint region is covered by a heat-shrinkable PEX casing installed over the HDPE jacket pipe using a mastic sealing tape. The present study considers a DN300 district-heating field-joint configuration as the target full-scale application case. The mastic tape used in the experiments was a commercial butyl-rubber-based sealing material employed in standard district-heating field-joint practice. In the DN300 configuration, the tape width was 160 mm and the circumferential length was approximately 500 mm. Since the same commercial product was applied consistently throughout the experimental program, variability attributable to the mastic material itself was limited within the present study. From a system-design perspective, the heating method should be selected by jointly considering trench geometry, compatibility with the PEX casing, and the feasibility of the equipment architecture and control scheme. Because joint casing installation in DH pipelines is performed in highly confined excavations, an automated heating module must deliver circumferential heating under strict geometric constraints while remaining mechanically robust against impacts, vibration, and self-weight during deployment and retrieval. In addition, when an enclosed heating chamber is formed around the casing, the chamber should prevent ingress of soil, dust, and moisture and reduce convective and radiative heat losses to the surrounding air and ground.

In a typical two-pipe (supply/return) configuration, the clearance between the outer HDPE jacket pipes is approximately 250 mm for DN 300 pipelines [24]. Because the heat-shrinkable joint casing has a larger outer diameter than the HDPE jacket, the minimum clearance between adjacent casings is expected to decrease to approximately 190 mm. This limited clearance defines the design envelope for the heating module, including the allowable heating angle and the stand-off distance, and constrains the circumferential placement of heater elements as well as the rotation radius required for an opening-and-closing mechanism. In addition, the confined installation environment introduces vibration and impact during handling; therefore, the module must tolerate repeated mechanical loading. Under these constraints, several non-contact options are difficult to implement reliably in the field: commercial infrared modules often require forced-air cooling and dedicated airflow passages, and quartz-tube emitters can be vulnerable to mechanical shock. Hot-air heating is highly sensitive to thermofluidic conditions (e.g., flow rate and local recirculation), which alters the convective heat-transfer coefficient and hence local heat flux, limiting repeatability. Induction heating, which relies on eddy-current losses in conductive media, cannot directly heat electrically insulating polymers such as PEX, HDPE, and butyl-rubber mastic tape. Therefore, the heating source for automated casing installation should deliver a stable and controllable surface heat flux at a short stand-off distance, sufficient to exceed the reference activation temperature defined in Section 2.1 and to initiate bottom anchoring at the 6 o’clock position before completing circumferential shrinkage.

**Figure 7:**
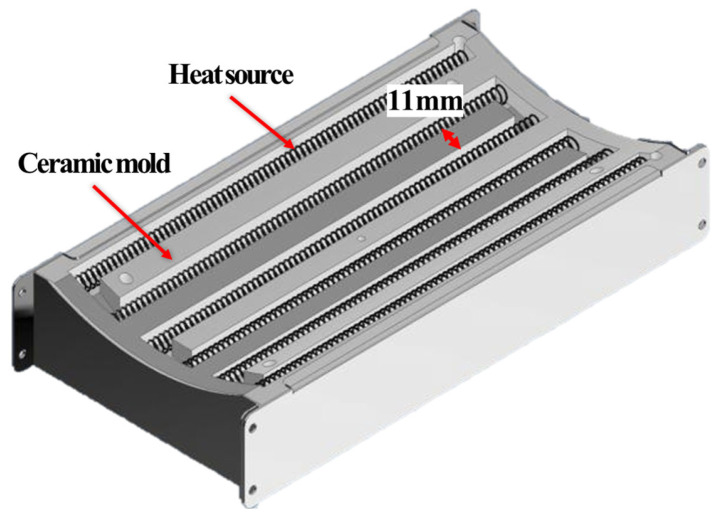
Structure of the heating module.

**Figure 8:**
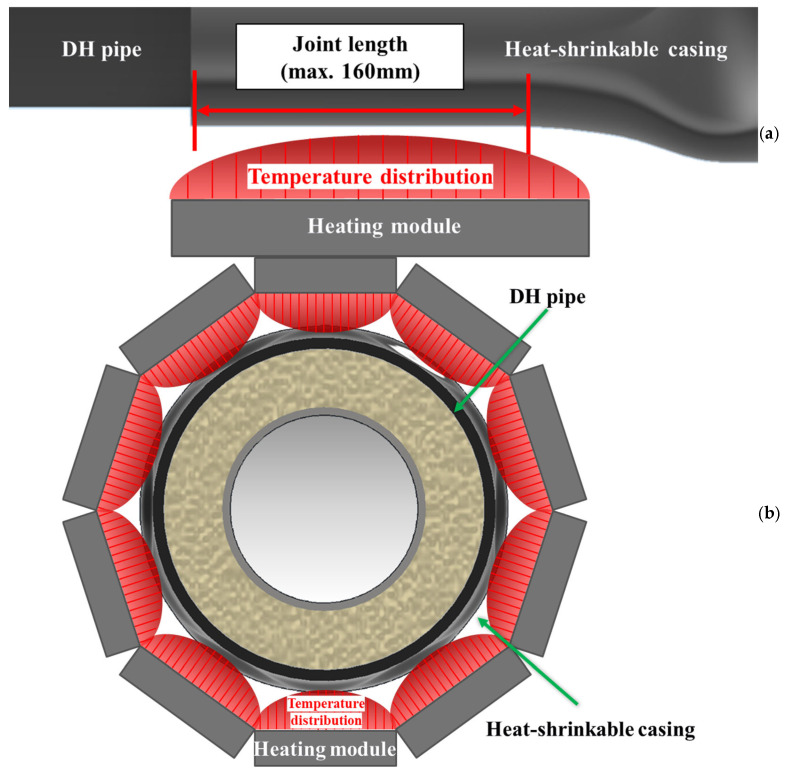
Conceptual representation of the heating module and temperature distribution: (**a**) Longitudinal direction; (**b**) Circumferential direction.

To satisfy the axial heating-span requirement and the circumferential heating-uniformity requirement simultaneously, the heating module was designed to deliver a controlled and repeatable casing-surface heat input over the required axial length while minimizing circumferential heat-flux non-uniformity. Figure 7 schematically illustrates the module architecture. As indicated in Figure 8a, an effective axial heating length of approximately 160 mm is required; accordingly, the module was configured to provide this heating span and to mitigate end underheating by reducing the axial temperature drop near both edges of the heated region, because insufficient end heating can lead to incomplete shrinkage and degraded sealing integrity. In addition, as illustrated in Figure 8b, the module must control the full 360° circumferential temperature distribution so as to suppress circumferential non-uniformity. Excessive circumferential non-uniform heating can induce premature local shrinkage, which can weaken initial fixation at the lower (6 o’clock) position and promote air-gap formation between the casing and the pipe surface. Therefore, the heater-array configuration, the curvature of the heating surface, and heat-flux equalization features were treated as key design parameters for controlling circumferential heat input. To meet these requirements, the module was designed to reach and maintain a casing-surface temperature exceeding the reference activation temperature (≥140 °C) within an enclosed heating chamber, and Table 1 summarizes the resulting design specifications (heater capacity, effective heating area, thermal response, and insulation layout) defined to satisfy these functional and thermal requirements.

### 3.2. Array of Heating Modules

The heating modules were arranged to maximize circumferential temperature uniformity of the heat-shrinkable casing during heating. To achieve uniform circumferential shrinkage, it is essential to maintain a nearly constant stand-off distance between each heating module and the casing surface, because variations in this gap directly affect the local heat flux distribution, shrinkage response, and interfacial bonding quality. In principle, a perfectly circular heater array would provide the most uniform heating conditions. However, the available envelope for hinges, stepper motors, and the opening-and-closing mechanism is limited, rendering a fully circular configuration impractical. In addition, mechanical interference between modules and manufacturing constraints restricts the implementation of a continuous circular structure. Therefore, polygonal heater arrangements were investigated to approximate a circular boundary while enabling practical equipment packaging.

(3) $$e(n)=R\times (1-\cos\frac{\pi }{n})$$

where R  is the radius of the casing and n  is the number of polygon sides. This expression represents the maximum inward deviation of a polygonal segment from the circular boundary, occurring at the midpoint of each segment. It derives from the fact that the perpendicular distance from the center of an inscribed regular polygon to its edges is R×cos(π/n); the difference between this distance and the circle radius yields the radial discrepancy. As n increases, cos(π/n) approaches 1, and e(n) decreases monotonically, indicating that polygons with more sides achieve a closer approximation to the circular casing profile.

**Figure 9:**
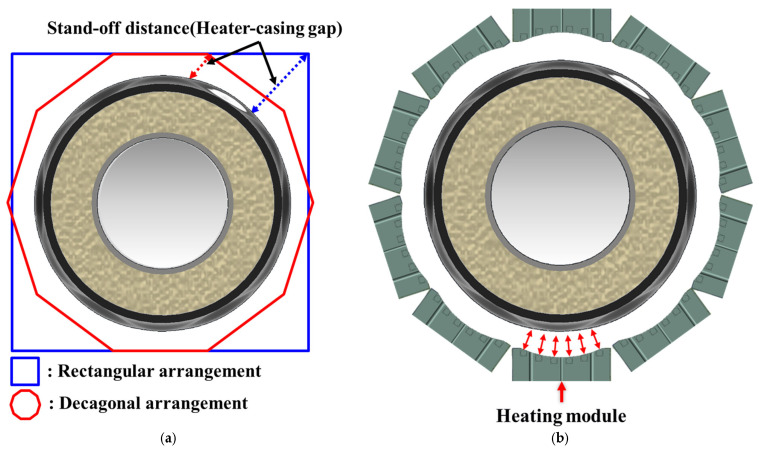
Conceptual illustration of the decagonal heater arrangement: (**a**) Comparison with a rectangular arrangement; (**b**) Heating module arrangement.

Figure 9a shows how the radial-gap error (e(n)) varies with the number of polygon sides (*n*) and confirms that the error decreases markedly when the configuration is changed from a rectangular frame to a decagonal frame. By contrast, the additional error reduction obtained by increasing the number of sides from 10 to 12 is relatively small and comparable to typical variations in casing outer diameter and assembly tolerances. Moreover, adopting 12 or more modules further reduces the width of each module and increases the number of interfaces associated with hinges, actuators, and sealing components, thereby complicating packaging within the restricted trench space and making it more difficult to maintain sufficient structural stiffness. Considering these geometric benefits and packaging constraints, this study adopts a decagonal heater array comprising ten modules as a practical trade-off. This configuration keeps the stand-off deviation sufficiently small for DN 300 casings while preserving adequate internal space for hinges, actuators, and sealing structures. During heating, thermal softening can induce casing deformation, and manufacturing and assembly tolerances can introduce outer-diameter variation; together, these effects can lead to local non-uniformity in the stand-off distance. To mitigate such non-uniformity, each heating module employs a curved ceramic heating surface that follows the casing curvature, thereby reducing local variations in the stand-off distance. Figure 9b illustrates the resulting decagonal arrangement of ten curved ceramic heating modules surrounding the casing. Each module is oriented such that its curved heating surface maintains an approximately constant stand-off distance from the casing surface. In particular, the lower region near the 6 o’clock position—where initial shrinkage and mastic-tape activation are most critical—is covered by multiple adjacent modules, whose curved profiles help maintain a locally uniform heater–casing gap near the 6 o’clock position, thereby reducing local circumferential heat-flux deficits. Compared with a conventional rectangular (flat) frame, this configuration decreases circumferential heat-flux non-uniformity, improving circumferential temperature uniformity during heating and the circumferential uniformity of the resulting shrinkage. Because the ceramic mold heaters used in this study are exposed-type radiative heaters, a short and well-controlled stand-off distance is required to deliver sufficient net heat flux to the casing surface. Even small increases in this gap can markedly reduce radiative heat transfer, making stand-off control a key design parameter governing PEX softening and the resulting shrinkage and bonding behavior. Accordingly, the inner diameter of the installation frame was determined by jointly considering casing-diameter variation, required structural stiffness, thermal expansion of the frame, and geometric constraints associated with module curvature and stable support for DN 300-class casings. Based on these considerations, the frame inner diameter was set to 542 mm. The axial length of each heating module was set to 240 mm, exceeding the required effective heating length (160 mm), to compensate for end effects (temperature drop near heater edges) and to ensure that the full target region reaches the reference temperature during operation.

**Figure 10:**
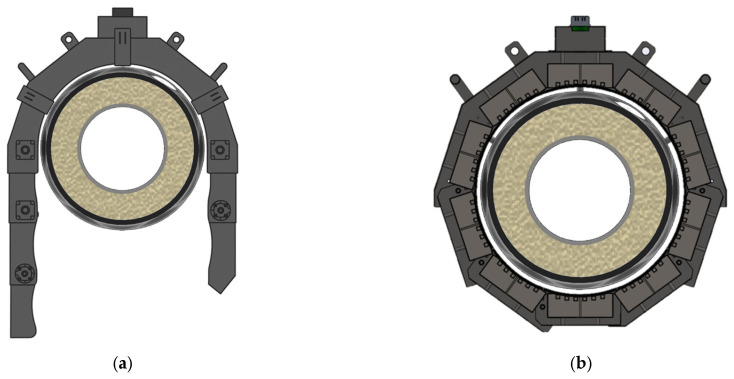
Equipment operation for joint casing installation: (**a**) Open state; (**b**) Closed state.

A mechanical opening-and-closing mechanism was integrated to deploy and enclose the heating-module assembly around the joint casing. In the open state (Figure 10a), the open-bottom configuration provides sufficient clearance to position the assembly without mechanical interference with the casing or surrounding components. After positioning and alignment, the modules are actuated to close and form a complete circumferential heating structure (Figure 10b). The motion is driven by a gear-reduced stepper motor, enabling precise, repeatable actuation and stable closure over repeated cycles. This controlled closing action minimizes module-to-module interference and misalignment, thereby maintaining a consistent stand-off geometry and improving repeatability under trench-constrained handling conditions.

**Figure 11:**
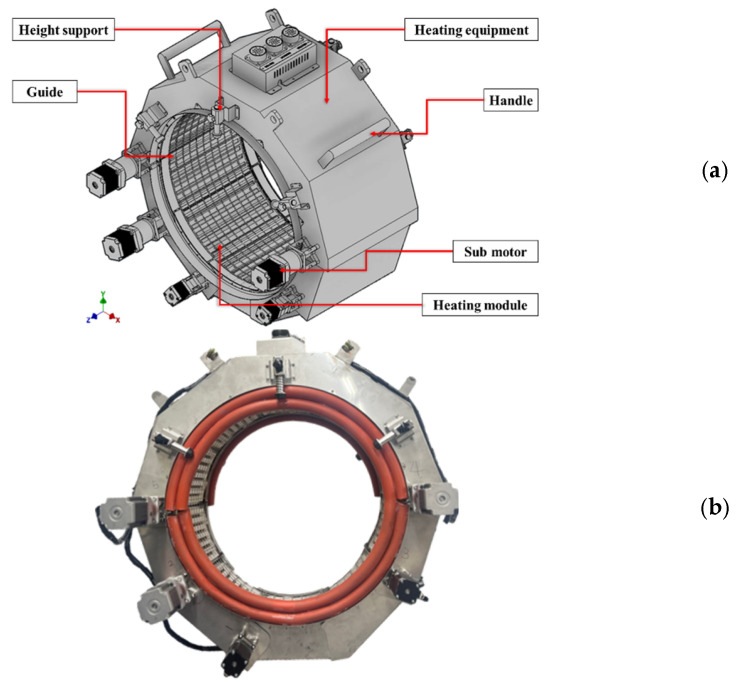
Overall configuration of the joint casing installation system: (**a**) Schematic configuration; (**b**) Developed equipment.

Figure 11 presents the overall configuration of the joint casing installation system. Figure 11a shows the schematic configuration, comprising a structural frame, heating modules, a guide system, a height-adjustment support, and an external control unit. The structural frame provides geometric rigidity for repeatable positioning of the casing relative to the heating modules, while the height-adjustment support enables accurate centering (coaxial alignment) with respect to the pipe axis. Figure 11b shows the developed equipment, in which curved heating modules are arranged to enclose the casing surface and form an enclosed heating chamber. Sealing elements integrated at the interfaces between adjacent modules limit ambient-air ingress and suppress convective heat loss within the chamber. By reducing heat loss at module junctions and mitigating edge cooling near module boundaries, these seals reduce circumferential heat-input non-uniformity, thereby improving circumferential uniformity of shrinkage and interfacial bonding.

Accordingly, the proposed heating-module design integrates (i) a decagonal layout, (ii) curved ceramic heating modules, (iii) a precision opening-and-closing mechanism, (iv) stand-off distance regulation, and (v) inter-module sealing structures. Together, these features provide the hardware basis for implementing the quantitatively defined circumferential heating procedure described in Section 4, including spatial sequencing and temporal power modulation. By reducing stand-off variation and suppressing heat loss at module junctions, the developed system is intended to reduce circumferential heat-input non-uniformity and improve the repeatability of shrinkage and interfacial bonding during heat-shrinkable casing installation in DH field joints.

### 3.3. Control of Heating Modules

The control strategy for the heating modules was formulated to deliver repeatable circumferential heating of the PEX casing while ensuring sufficient thermal activation of the mastic tape. The strategy was developed by considering (i) the through-thickness heat-transfer behavior of the heat-shrinkable casing, which is limited by its low thermal conductivity [24,25,26], (ii) the confined installation conditions typical of buried DH trenches, and (iii) the heating characteristics of the ceramic mold heaters used in this study. Accordingly, the control objectives are to limit circumferential heat-input non-uniformity, avoid localized overheating, and achieve reproducible, operator-independent installation quality. To this end, the spatial progression of manual gas-torch heating used in field practice was used to define a four-stage bottom-up circumferential heating sequence under quantitative control. First, the lower region of the casing (6 o’clock position) is heated to establish an initial anchor to the pipe surface. Second, the mid-height region is heated to extend shrinkage from the lower anchor toward the upper region. Third, the upper region is heated to complete circumferential shrinkage. This bottom-up process suppresses downward sagging associated with thermal softening and promotes early tack formation of the mastic tape. Finally, a brief circumferential equalization stage is applied by activating all modules simultaneously to reduce residual circumferential temperature non-uniformity. In this way, the automated system reproduces the circumferential progression of manual torch heating while enabling quantitative, stage-wise control of heating duration and heater output power. The temperature of each module is monitored using sensors embedded near the heater surface, and the output is regulated to track a prescribed setpoint temperature defined based on the thermal-transition characteristics of the PEX casing. This closed-loop control mitigates disturbances arising from environmental heat loss, casing-thickness variation, and fluctuations in the stand-off distance under field conditions. In addition, together with the curved heater surfaces, the positioning mechanism maintains a nearly constant and uniform stand-off distance after closure despite allowable casing-diameter deviations and prevents module-to-module interference, both of which are critical for delivering a consistent surface heat flux to the casing. In summary, the proposed control system integrates (i) a directional heating process, (ii) target-temperature feedback control, and (iii) stand-off distance regulation to compensate for casing-diameter variation. By reducing the operator-dependent nature of torch-based heating, the proposed automated system improves the uniformity and repeatability of heat-shrinkable casing installation.

## 4. Performance Test with Automated Installation System

### 4.1. Evaluation of Heating Process

**Figure 12:**
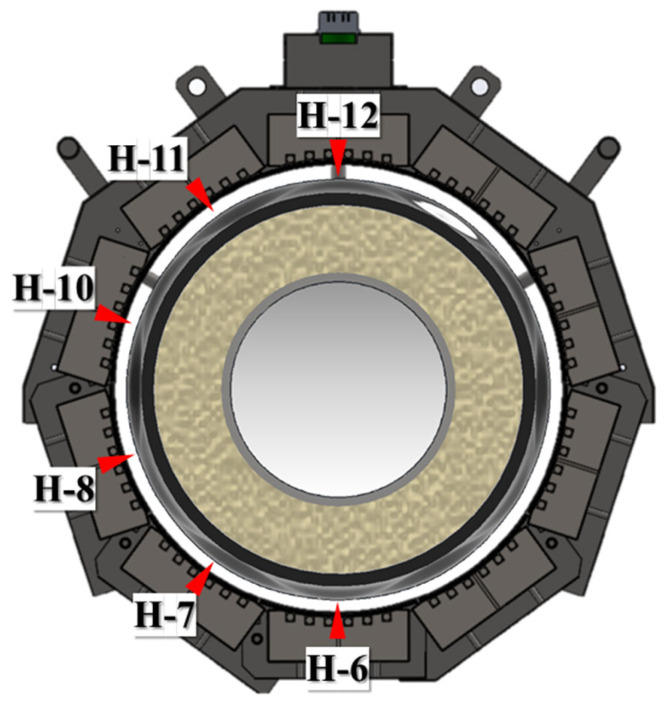
Locations of the outer-surface temperature measurement points (H-6 to H-12).

Figure 12 defines the locations and labeling scheme of the outer-surface measurement points (H-6 to H-12) used in this section. The points were placed 10 mm from the axial edge to minimize axial end effects and edge cooling. Contact K-type thermocouples were mechanically secured to the casing surface using a steel pin to ensure stable thermal contact during the rapid temperature rise. All measurement points were located within the circumferential coverage region of the joint casing installation system, enabling evaluation of the $T_{outer}$ response in the module-facing (directly heated) region. The applied heating times for each position during the heating sequence are summarized in Table 2.

**Figure 13:**
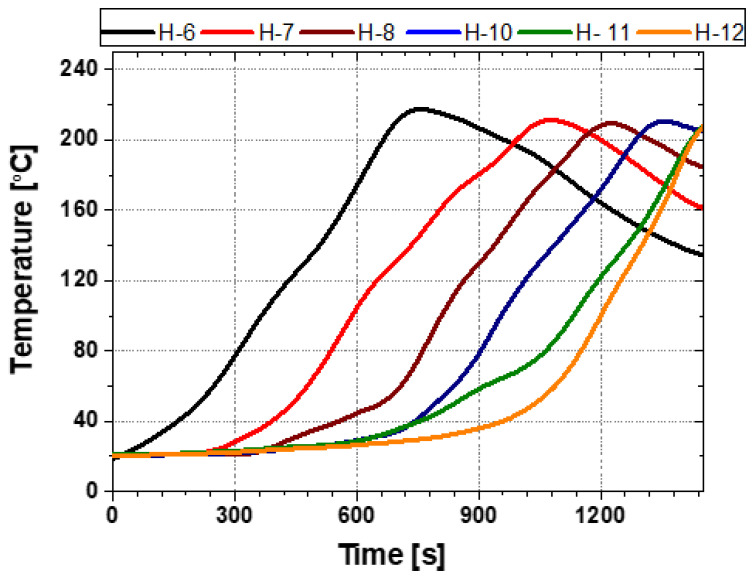
Temperature histories on the outer-surface of heat-shrinkable casing.

As shown in Figure 13, the $T_{outer}$ at all measurement points increased steadily with time and exceeded the reference activation temperature (approximately 140 °C) for the PEX heat-shrinkable casing. These results indicate that, under the tested single-output (100%) circumferential sequential-heating operation, the automated installation system can raise the casing $T_{outer}$ into the activation range required to initiate shrinkage. In addition, the temperature–time responses at the module-facing measurement points exhibited only limited scatter, indicating relatively consistent circumferential outer-surface heating within the directly heated region under the present operation. However, the $T_{outer}$ alone does not guarantee that the inner surface (and the casing–mastic interface) simultaneously reaches the activation temperature. Because PEX has inherently low thermal conductivity, through-thickness heat conduction is slow, resulting in a time lag between the outer- and inner-surface temperature rise. This limitation becomes more pronounced in regions where the local heat input is reduced or heat losses are elevated, such as near module boundaries and locations affected by axial end cooling.

**Figure 14:**
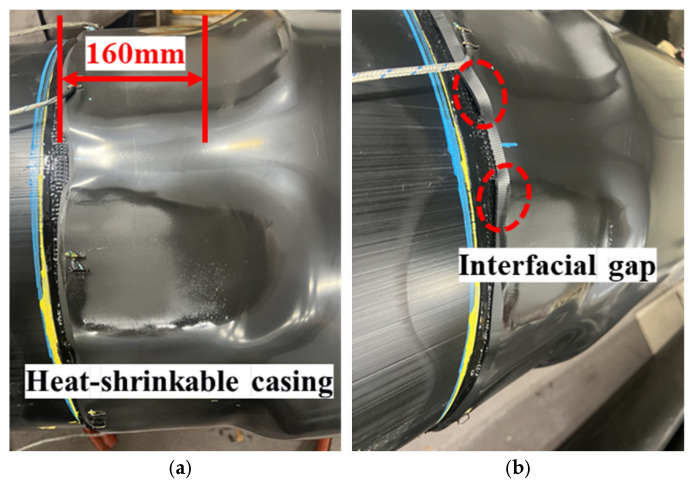
Outer shape of bonded casing: (**a**) Longitudinal view; (**b**) Side view.

The shrinkage behavior obtained under the single-output (100%) circumferential sequential-heating operation is summarized in Figure 14. The longitudinal view in Figure 14a indicates that the heated zone achieved the intended effective axial shrinkage length (≈160 mm), suggesting that axial shrinkage within the module-coverage region is sufficiently activated under this condition. In contrast, the side view in Figure 14b reveals that circumferential shrinkage is not fully uniform: certain circumferential sectors conform tightly to the outer HDPE jacket, whereas other sectors retain a larger apparent diameter and exhibit reduced conformity, particularly near the axial edges. This circumferential non-uniformity is consistent with spatial variations in the delivered surface heat flux. For example, locally increased heater–casing stand-off distance and less favorable radiative exposure reduce the net heat input, slowing the $T_{outer}$ rise and, more critically, delaying through-thickness heat penetration to the inner surface and the casing–mastic interface. Consequently, despite the outer surface exceeding the reference activation temperature, thermally disadvantaged circumferential sectors may fail to satisfy the inner-surface/interface activation requirement within the applied heating time for each position (Table 2). These observations motivate the development of a criterion-based heating process that explicitly accounts for through-thickness thermal lag and circumferential heat-flux non-uniformity, rather than relying solely on $T_{outer}$ rise under a fixed maximum-output condition.

Taken together, the results in Figure 13 and Figure 14 indicate that circumferential sectors experiencing insufficient heating can compromise sealing integrity and show that a fixed single-stage, single-direction operation at the maximum output is insufficient to achieve consistent shrinkage over the full casing circumference. Accordingly, the present test serves as an initial performance assessment of the automated joint casing installation system and provides an experimental basis for developing a heating strategy that redistributes circumferential heat input. This strategy is implemented through the multi-stage circumferential heating process described in Section 4.2.

### 4.2. Derivation of a Criterion-Based Heating Process

**Figure 15:**
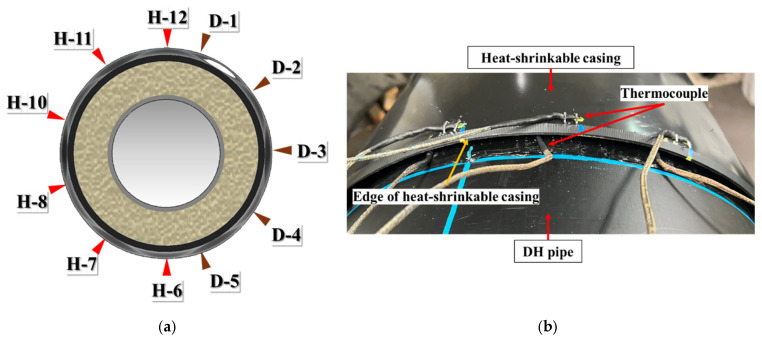
Temperature measurement: (**a**) Locations of measurement points on the heat-shrinkable casing; (**b**) example of thermocouple attachment on the heat-shrinkable casing.

To establish an appropriate heating procedure, supplementary heating tests were conducted with simultaneous temperature measurements on the outer and inner surfaces of the heat-shrinkable casing. The resulting data were then used to derive the heating sequence, considering the thermal criteria and through-thickness thermal lag described in Section 2.1. The thermocouple arrangement is schematically shown in Figure 15a. K-type thermocouples were attached to the outer surface at locations 10 mm from the axial edge to monitor $T_{outer}$, which was used as the practical design variable for development of the heating procedure. To evaluate through-thickness thermal penetration to the location of the casing–mastic interface, additional thermocouples were installed on the inner surface of the casing. The paired outer- and inner-surface temperature measurements in this study enabled quantitative assessment of through-thickness heat-transfer effectiveness and identification of the activation criterion. However, the observed outer–inner temperature relationship was used only as an experimental basis for establishing the heating criteria, rather than to formulate a generalized predictive correlation for field application, because this relationship depends on the specific heating configuration and transient installation condition. Figure 15b shows the thermocouple attachment on the casing outer surface. Sensors were placed both in the zone directly facing the ceramic heating modules and in the circumferential regions between adjacent modules (inter-module gaps), so that the measurements captured temperature gradients between directly heated areas and reduced-heat-input gap regions representative of circumferential thermal non-uniformity during automated heating.

**Figure 16:**
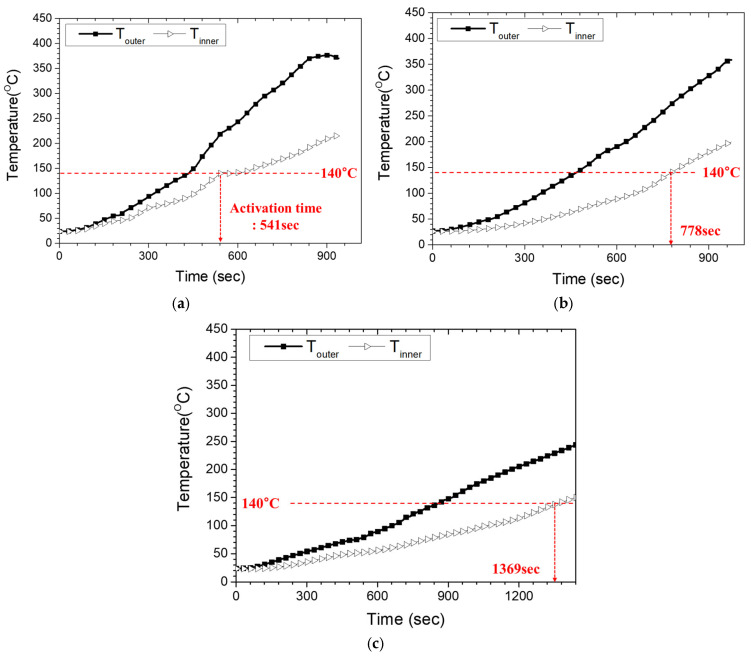
Measured outer- and inner-surface temperature histories of the casing under different heater power settings: (**a**) 100%; (**b**) 75%; (**c**) 50%.

**Figure 17:**
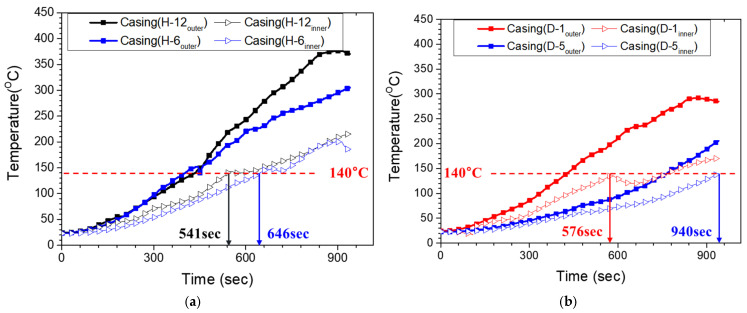
Experimental temperature–time histories measured of circumferential positions: (**a**) module-aligned positions; (**b**) gap-aligned positions (between adjacent heating modules).

To quantify the relationship between the outer- and inner-surface temperatures, additional laboratory tests were performed by attaching thermocouples to both surfaces of the casing specimen at matching circumferential locations. The specimen was heated using the developed joint casing installation system under controlled operating conditions at power settings of 100%, 75%, and 50%. Figure 16a–c compare the corresponding inner- and $T_{outer}$ histories. At 100% output, the $T_{inner}$ reached the reference activation temperature (140 °C) after 541 s, at which time the $T_{outer}$ was 218.2 °C. At 75% output, the inner surface reached 140 °C after 778 s, while the corresponding $T_{outer}$ increased to 272.7 °C, approaching the maximum allowable $T_{outer}$ (≈280 °C) defined in Section 2.1. At 50% output, the inner surface reached 140 °C after 1369 s, and the corresponding $T_{outer}$ was 231.9 °C. Notably, although the 50% case required the longest heating time, its $T_{outer}$ at inner-surface activation remained substantially lower than that of the 75% case. Because the comparison is made at the time when the inner surface reaches 140 °C, the $T_{outer}$ reflects not only the output level but also the time required to satisfy the inner-surface criterion. In the 75% case, the longer time to inner-surface activation allows the outer surface in the directly heated zone to continue rising toward a higher quasi-steady level, whereas the 50% case lowers the surface heat flux sufficiently that the outer surface remains substantially cooler even over the longer heating duration. Therefore, 50% was selected for the reduced-output stage to provide additional dwell time for through-thickness and lateral heat conduction while limiting further increases in $T_{outer}$. Overall, the 100% output condition achieved the shortest time to inner-surface activation while maintaining a large margin to the degradation threshold. These experimentally observed trends provide a practical basis for defining the heating procedure; however, the physical origin of the circumferential temperature non-uniformity should be interpreted with caution because multiple mechanisms are coupled during heating, including geometric shielding, inter-module gap effects, convective heat loss, heater–casing distance variation, and thermal contact at the casing–mastic–pipe interface [36,37,38]. Because these effects evolve simultaneously during heating and shrinkage, rigorous factor-by-factor quantitative separation is not straightforward under the present installation condition. Therefore, the present study focused on establishing practical heating criteria from the experimentally observed response. Based on these results, an $T_{outer}$ target of approximately 230 °C in the directly heated zone was adopted as a conservative design criterion to reliably achieve inner-surface activation while remaining comfortably below the ~280 °C threshold. For the inter-module gap region, which exhibits consistently lower outer-surface temperatures than directly heated locations (Figure 17), a minimum target $T_{outer}$ of 205 °C was adopted as a conservative lower bound. This threshold was experimentally verified to be sufficient to achieve complete shrinkage and interfacial bonding in the gap region without observable surface damage.

The circumferential temperature non-uniformity between the directly heated zone and the inter-module gap region was quantified. Figure 17a shows representative $T_{outer}$ histories at directly heated positions (e.g., H12 and H6), where the temperature rises rapidly during the early stage due to radiative heating from the ceramic mold heaters. At these locations, the $T_{outer}$ exceeds the design threshold, suggesting that the local heating conditions are favorable for activation of the interfacial mastic layer. In contrast, Figure 17b shows the $T_{outer}$ histories at the inter-module gap locations (D1 and D5), which exhibit a lower initial heating rate and a delayed approach to the design threshold compared with the directly heated positions. Throughout heating, the $T_{inner}$ in the gap region remained approximately 10–15 °C lower than that in the directly heated zone. Because this region receives reduced direct radiative heating and relies primarily on lateral heat conduction from neighboring heated areas, it is prone to delayed shrinkage and mastic activation relative to the directly heated regions. These observations indicate that a single-stage fixed-output condition is insufficient to fully equalize the circumferential temperature field; therefore, the heating process should incorporate additional control actions targeting the inter-module gap regions.

To address this limitation, a two-stage heating strategy was developed based on the measured temperature responses. In the first stage, all heating modules operate at 100% output to rapidly raise the $T_{Outer}$ in the directly heated regions to a level sufficient to drive inner-surface activation, thereby initiating shrinkage and interfacial bonding in the module-facing areas. Simultaneously, the remaining circumferential regions are preheated from ambient temperature and begin to warm up. Because reduced-heat-input regions such as the inter-module gaps are heated primarily by lateral conduction from adjacent modules, their temperature rise lags behind that of the directly heated regions. Accordingly, in the second stage, the output of all heating modules is uniformly reduced to 50%. This reduced output suppresses further increases in $T_{Outer}$ in regions that have already reached the required outer-surface heating level, while providing extended dwell time for heat to diffuse circumferentially and through the wall thickness into previously underheated regions. As a result, the temperature difference between the directly heated and inter-module gap regions decreases, enabling all circumferential positions to satisfy the thermal conditions required for shrinkage and mastic activation. From a through-thickness heat-conduction perspective, the two-stage heating strategy addresses two competing thermal requirements associated with PEX. First, the inner-surface temperature ($T_{inner}$)—and, critically, the casing–mastic interface—must reach the reference activation temperature (≈140 °C) to enable shrinkage and sufficient mastic activation for bonding. Second, the $T_{Outer}$ must remain below the thermal-degradation threshold (≈280 °C), beyond which prolonged exposure may initiate material degradation. Under the 100% output condition, $T_{Outer}$ in the directly heated zone rises rapidly (typically to approximately 230 °C), while $T_{inner}$ can reach the activation temperature within a relatively short time without exceeding the defined degradation limit. In the subsequent 50% output stage, the reduced surface heat flux limits further increase in $T_{Outer}$ in the directly heated regions while allowing lateral and through-thickness conduction to raise temperatures in reduced-heat-input regions (e.g., inter-module gaps) toward the required activation conditions. Accordingly, the experimentally derived two-stage sequence is designed to ensure that each circumferential location satisfies the dual constraint of inner-surface activation ($T_{inner}$ ≥ 140 °C) while maintaining the outer-surface temperature below the degradation threshold ($T_{Outer}\;$ < 280 °C), rather than enforcing perfectly uniform temperatures. This criterion-based control improves the reliability of shrinkage and interfacial bonding over the full circumference.

The circumferential thermal non-uniformity observed in the present study should be interpreted as the combined outcome of multiple coupled mechanisms, including geometric shielding, inter-module gap effects, convective heat loss, heater–casing distance variation, and evolving thermal contact at the casing–mastic–pipe interface. The difficulty of quantitatively separating these effects is inherent to the present installation condition because the casing geometry and boundary conditions change continuously during heating as the heat-shrinkable casing softens and contracts. Accordingly, the present results are used to establish experimentally grounded heating criteria and a practical heating sequence under representative installation conditions, rather than to provide a factor-by-factor decomposition of individual design and operational contributions.

**Figure 18:**
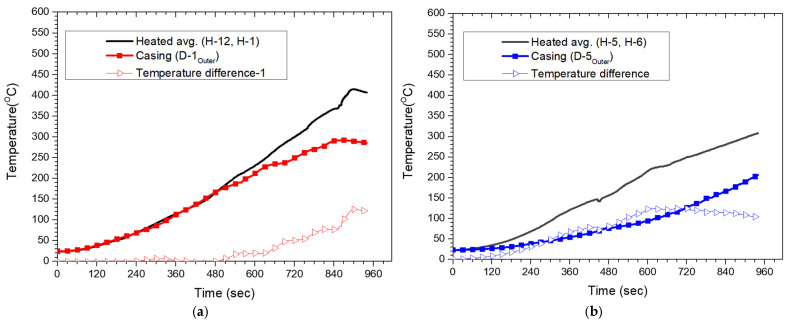
Outer-surface temperature difference between directly heated positions and the inter-module gap position: (**a**) H-12, D-1, and H-1; (**b**) H-5, D-5, and H-6.

To quantify the circumferential thermal non-uniformity observed in Figure 17 more directly, the outer-surface temperature difference between the directly heated positions and the corresponding inter-module gap position was evaluated as a function of time, as shown in Figure 18. In each panel, the solid line denotes the average outer-surface temperature of the directly heated positions, the square-marked line denotes the outer-surface temperature at the corresponding inter-module gap position, and the triangle-marked line denotes the temperature difference between these two values. In Figure 18a, the temperature difference between the directly heated positions and D-1 increased progressively during heating and reached a maximum of approximately 125 °C. A similar trend is observed in Figure 18b, where the temperature difference between the directly heated positions and D-5 also increased substantially and reached a maximum of approximately 125 °C. These results confirm that the inter-module gap positions remained thermally disadvantaged relative to the adjacent directly heated positions throughout heating. Therefore, the heating procedure should account not only for the temperature response in directly heated regions but also for the thermal deficit inter-module gap regions.

**Figure 19:**
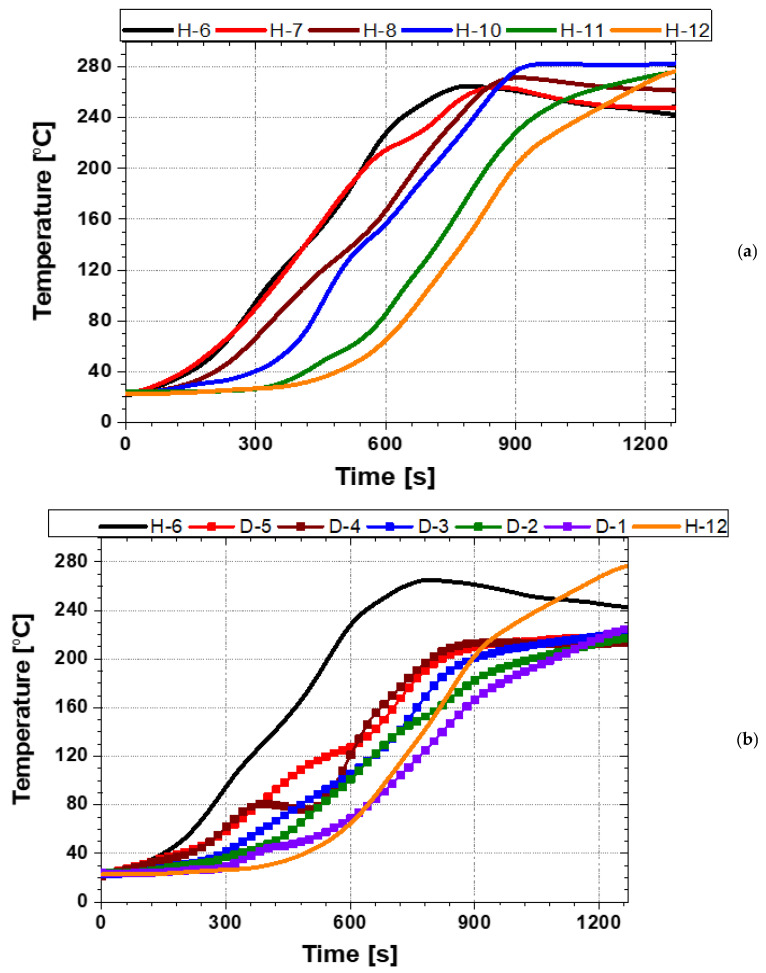
Outer-surface temperature histories of the heat-shrinkable casing: (**a**) module-aligned positions; (**b**) gap-aligned positions (between adjacent heating modules).

The proposed strategy was experimentally validated through a full-circumference (360°) heating test using the two-stage sequence. Figure 19a shows the $T_{outer}$ response in the directly heated zone. During the first stage (100% output), the temperature stabilized at approximately 230 °C, and during the subsequent second stage (50% output) it remained below the degradation threshold (≈280 °C). Here, 205 °C denotes the minimum $T_{outer}$ required in the inter-module gap region to ensure inner-surface activation ($T_{inner}$ ≥ 140 °C). It was obtained by subtracting the observed 10–15 °C gap-region deficit from the measured condition $T_{outter}$ = 218.2 °C at $T_{inner}$ = 140 °C. Figure 19b presents the corresponding response in the inter-module gap region. All measurement points exceeded the minimum gap-region outer-surface criterion (205 °C) and remained above this threshold during the reduced-output stage, indicating that sufficient heat input was delivered even in the inter-module gap region. Overall, these results confirm that heat transfer from adjacent modules can provide adequate activation conditions in the inter-module gap regions under the proposed two-stage sequence. The adopted threshold of 205 °C therefore represents a conservative outer-surface criterion that incorporates the observed circumferential temperature deficit in the inter-module gap region and provides a practical safety margin for ensuring inner-surface activation.

By correlating the measured circumferential temperature distributions with the observed shrinkage behavior, the required stage-wise heating durations at each circumferential location were determined for the 100% and 50% output stages. These experimentally derived conditions were consolidated to define the final operating process summarized in Table 3. The resulting two-stage circumferential heating schedule applies to a high-power preheating stage (100% output) followed by a reduced-power soaking stage (50% output) at each circumferential position. The schedule is embedded within the bottom-up circumferential progression; thus, the start time of the 100% stage varies by position, while the subsequent 50% stage serves as a soak/equalization period until completion.

## 5. Results and Discussion

To assess whether the proposed thermal criteria and two-stage heating sequence improve shrinkage completeness and installation uniformity, the post-installation surface conditions of PEX heat-shrinkable casings produced by the controlled circumferential heating procedure were compared with those produced by conventional manual gas-torch heating. For the controlled procedure, the heaters were operated according to the two-stage sequence summarized in Table 3. For manual installation, experienced operators adjusted torch heating to (i) achieve a representative mid-length outer-surface temperature commonly targeted in field practice and (ii) complete the installation within a comparable practical time frame. After heating, all specimens were air-cooled to ambient temperature prior to visual inspection.

**Figure 20:**
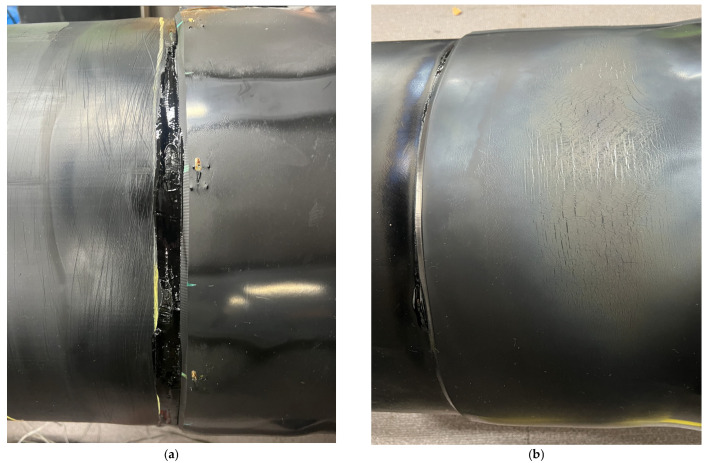
Heating result of heat-shrinkable casing: (**a**) Installed using the automated system; (**b**) Installed using the manual torch method.

Figure 20a shows a representative casing installed using the controlled procedure. Over the heated axial region, the casing exhibits a smooth surface and a consistent post-shrinkage outer diameter, without pronounced wrinkles or visibly under-shrunk bands near the axial edges. Figure 20b shows a representative casing installed by manual torch heating. While the manual specimen can also appear acceptable by routine visual inspection, it more frequently exhibits local surface undulations and edge-related irregularities, which are consistent with localized over- and under-heating during manual operation in confined trench conditions. Importantly, because surface appearance does not directly quantify interfacial activation and bonding continuity at the casing–mastic interface, these macroscopic observations are insufficient to distinguish latent bonding variability. Therefore, quantitative shear testing and post-failure observations were performed to evaluate interfacial bonding performance in the following sections.

**Figure 21:**
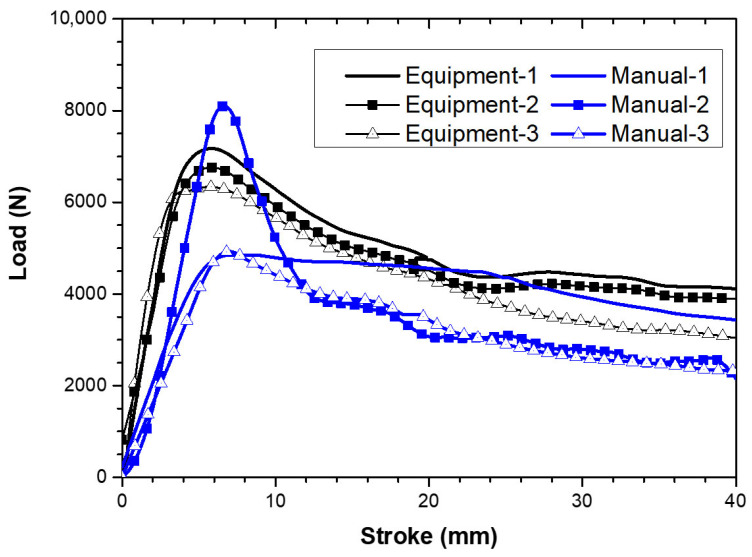
Comparison of bonding strengths between automated joint casing installation system and manual method.

To quantify interfacial bonding performance, shear tests were conducted on casings installed using the controlled circumferential heating procedure and by manual gas-torch heating. Figure 21 compares the load–displacement responses. All specimens exhibit a distinct peak load followed by post-peak softening, indicating initiation of interfacial damage and progressive debonding after the peak.

The specimens installed using the controlled procedure show highly consistent peak loads of approximately 7000 N, with closely overlapping post-peak responses, demonstrating repeatable bonding performance under the proposed thermal criteria and two-stage heating sequence. In contrast, the manually heated specimens show substantial scatter. The measured peak loads were 4888 N (Manual-1), 8370 N (Manual-2), and 4958 N (Manual-3), yielding a mean of 6072 N and a standard deviation of 1990 N (coefficient of variation, 32.8%). When referenced to the controlled condition (~7000 N), the manual condition exhibits a lower mean strength level (≈13% lower) and markedly reduced repeatability. These results indicate that manual torch heating can occasionally produce high apparent strength, but the outcome is inconsistent, likely reflecting localized over- and under-heating and the absence of quantitative constraints during in situ installation.

**Figure 22:**
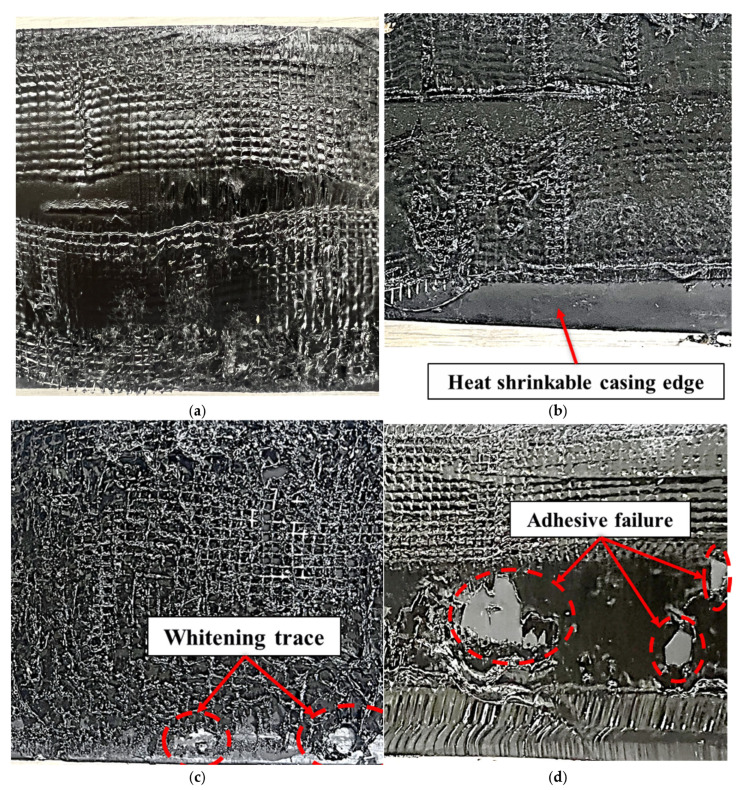
Casing–mastic tape interface after shear testing: (**a**) Automated joint casing installation system; (**b**) Manual-1; (**c**) Manual-2; (**d**) Manual-3.

Post-shear observations provide insight into the origin of the strength scatter observed in Figure 20. Figure 22 compares the dominant failure features for specimens installed using the controlled circumferential heating procedure and by manual torch heating. In the controlled specimens, the mastic tape remains continuously adhered over the overlap region, and failure occurs predominantly as cohesive failure within the mastic layer. This failure mode suggests that interfacial adhesion at the casing–mastic interface exceeded the intrinsic cohesive strength of the mastic, which is consistent with sufficiently complete shrinkage and adequate thermal activation of the interface under the proposed thermal criteria. In contrast, the manually heated specimens exhibit multiple non-ideal features that are consistent with localized over- and under-heating. Manual-1, which exhibited a low peak load, shows insufficient adhesion near the axial edge region, implying a reduced effective bonded area and incomplete interfacial development in a location where stress concentration can occur. Manual-2, which exhibited the highest peak load, shows local whitening and evidence consistent with unintended local fusion/direct bonding between the steel service pipe and the heat-shrinkable casing. Although such localized overheating can increase the apparent peak load, it deviates from the intended sealing mechanism and may be undesirable for long-term performance because it can introduce local stiffness mismatch and residual stresses under thermal cycling. Manual-3 shows localized adhesive failure at multiple circumferential positions, indicating circumferentially non-uniform bonding.

Overall, the shift from frequent interfacial or mixed-mode failures (manual) to predominantly cohesive mastic failure (controlled) supports that the controlled procedure more consistently achieves through-thickness thermal penetration and interface activation around the full circumference, thereby reducing latent bonding non-uniformity that may not be detectable by visual inspection alone. The failure analysis in this study was limited to macroscopic visual examination of the post-shear fracture modes to identify the dominant failure mechanism of the bonded joint. Figure 22 therefore represents qualitative observations of fracture behavior rather than a detailed microstructural characterization of the adhesion interface. Residual stresses associated with localized overheating were not quantitatively evaluated because the heating conditions were controlled to remain below the thermal degradation threshold of the PEX casing.

In addition to bonding reliability, practical deployment requires comparison of installation time, repeatability, and operational safety between manual torch heating and the proposed controlled heating method. Under the present DN 300 test conditions, the controlled circumferential heating procedure required approximately 1130 s per casing, whereas manual torch heating required approximately 300 s per casing. Although manual heating offers a shorter cycle time due to the highly localized heat input of direct flame application, it is also associated with substantial operator-dependent variability in local heating conditions. Because the local heat input during manual operation depends strongly on flame angle, stand-off distance, local dwell time, and circumferential movement, the resulting casing-surface heating exhibits considerable spatial and temporal variation. Accordingly, a direct temperature-history comparison between manual torch heating and the automated heating system is of limited representativeness, and a rigorous comparison of total energy consumption was not attempted in the present study. Instead, the comparison was made in terms of practically relevant installation outcomes, including bonding performance, failure mode, installation time, repeatability, and field operability. As discussed in Section 4.2, the shorter cycle time of manual heating was accompanied by substantially higher bonding variability and a greater risk of localized overheating and underheating, particularly near axial edges and circumferentially occluded regions, whereas the controlled circumferential heating procedure, although slower, provided improved repeatability, reduced strength scatter, and a mechanism-consistent failure mode characterized predominantly by cohesive failure in the mastic layer. These results indicate a trade-off between processing time and installation reliability and suggest that the proposed thermal criteria and two-stage heating sequence provide a more standardized and reproducible basis for installation under confined trench conditions.

From a quality-control perspective, the key implication is that visual inspection alone may not detect latent non-uniform bonding; therefore, a procedure that improves repeatability can reduce the uncertainty in field outcomes. Although the present controlled procedure is slower, it produces a mechanism-consistent failure mode (predominantly cohesive failure in the mastic) and markedly reduces strength scatter, which can lower the probability of interfacial weak spots that may govern long-term sealing performance. In confined trench environments, the use of an open flame also introduces operational hazards and may be restricted by site regulations, whereas controlled heating can reduce worker exposure to direct flame and hot-gas jets.

Overall, the results indicate a trade-off between processing time and installation reliability. The proposed thermal criteria and two-stage heating sequence provide a basis for standardized installation that prioritizes repeatable interfacial activation, while further study is needed to quantitatively evaluate the individual contributions of the coupled factors affecting circumferential heating non-uniformity under representative installation conditions.

## 6. Conclusions

This study establishes experimentally grounded thermal criteria and a reproducible heating procedure for heat-shrinkable crosslinked polyethylene (PEX) joint casings installed under field-constrained irradiation conditions. DSC measurements were used to define a reference activation condition (approximately 140 °C), while installation heating was additionally constrained by an upper bound on the allowable $T_{outer}$ to avoid thermal damage (≈280 °C). Full-scale temperature measurements revealed circumferential heat-input non-uniformity, automated induction reduced-temperature regions at module-gap/occluded locations (approximately 10–15 °C lower than directly irradiated regions), and a pronounced through-thickness thermal lag arising from the low thermal conductivity of PEX. Based on these observations, a two-stage heating sequence was derived to satisfy dual constraints—inner-surface activation and an $T_{outer}$ upper bound—while mitigating circumferential temperature differences. Comparative joint tests demonstrated that the controlled procedure yields continuous mastic-tape bonding with predominantly cohesive failure and markedly reduced scatter in bonding strength, whereas manual torch heating remains highly sensitive to localized over- and under-heating.

The main contributions of this work are as follows:

(1) Establishment of polymer-specific thermal criteria for heat-shrink installation by coupling DSC-defined activation with full-scale, through-thickness temperature measurements.

(2) Experimental quantification of circumferential thermal non-uniformity and identification of its interaction with through-thickness thermal lag, which motivates stage-wise power modulation rather than single-stage heating.

(3) Validation of a two-stage heating sequence that improves the repeatability of interfacial bonding and shifts failure toward mastic cohesive failure, thereby strengthening the process–performance link for heat-shrinkable polymer casing systems.

The present thermal criteria and heating sequence were calibrated for DN 300 joints under a specific combination of casing geometry, trench constraints, and environmental conditions. Extension to other pipe diameters, casing thicknesses, trench geometries, or ambient conditions will require retuning of key parameters (e.g., heater power and stage durations) while maintaining the same dual-constraint framework. Further work should also address productivity improvements and a systematic techno-economic assessment, while ensuring that any time reduction does not violate the established thermal criteria.

## Figures and Tables

**Table 1:** Design specifications of the heating module for the heat-shrinkable casing system.

Category	Parameter	Description
Heater geometry	Module shape	Curved ceramic mold heater
	Surface curvature	Applied to ensure uniform heating distance
	Heating surface length	160 mm
Thermal performance	Target heating temperature	≥140 °C (inner surface/interface criterion)
	Achieved peak temperature	280 °C rated heater surface temperature
Electrical characteristics	Rated power (W)	11.5 kW
Control & Integration	Temperature sensing	Internal thermocouple
	Control method	Independent module control via controller

**Table 2:** Heating conditions for performance test.

Position	Heating Time [s]
H-12	448–983
H-11	366–983
H-10	309–915
H-8	192–908
H-7	0–763
H-6	0–690

**Table 3:** Two-stage heating sequence.

Position	100% Output [s]	50% Output [s]
H-12	370–870	870–1130
H-11	310–870	870–1130
H-10	210–870	870–1130
H-8	60–840	840–1130
H-7	0–780	780–1130
H-6	0–720	720–1130

## Data Availability

The data presented in this study are not publicly available, but may be available from the corresponding author upon reasonable request.
